# Microseismic records classification using capsule network with limited training samples in underground mining

**DOI:** 10.1038/s41598-020-70916-z

**Published:** 2020-08-18

**Authors:** Pingan Peng, Zhengxiang He, Liguan Wang, Yuanjian Jiang

**Affiliations:** 1 School of Resources and Safety Engineering, Central South University, Changsha, 410083 China; 2 Digital Mine Research Center, Central South University, Changsha, 410083 China

**Keywords:** Civil engineering, Geology, Seismology

## Abstract

The identification of suspicious microseismic events is the first crucial step in microseismic data processing. Existing automatic classification methods are based on the training of a large data set, which is challenging to apply in mines without a long-term manual data processing. In this paper, we present a method to automatically classify microseismic records with limited samples in underground mines based on capsule networks (CapsNet). We divide each microseismic record into 33 frames, then extract 21 commonly used features in time and frequency from each frame. Consequently, a 21 × 33 feature matrix is utilized as the input of CapsNet. On this basis, we use different sizes of training sets to train the classification models separately. The trained model is tested using the same test set containing 3,200 microseismic records and compared to convolutional neural networks (CNN) and traditional machine learning methods. Results show that the accuracy of our proposed method is 99.2% with limited training samples. It is superior to CNN and traditional machine learning methods in terms of Accuracy, Precision, Recall, F1-Measure, and reliability.

## Introduction

Underground engineering causes disturbances in the stress state of the rock mass, leading to a large number of microseismic events^1^. By post-processing these records (e.g., P-wave arrival picking^2^, event location^3^, and source parameter calculation^4–6^), the mechanical state of the corresponding rock mass can be adequately reflected, which is beneficial especially for disaster early warning in underground mining^7–9^. However, in the underground mining process, the microseismic monitoring system often receives interference from blasting operations, ore extraction, mechanical operations, high voltage cables, and magnetic fields^10^. Therefore, quickly and accurately identifying microseismic records from a large number of suspicious records is a crucial task. Currently, the classification of suspicious microseismic records depends on the visual scanning of waveforms by experienced analysts^11^. However, manual classification of microseismic records is a time-consuming, tedious task that is easy to bring into subjective opinions. For these reasons, automatic classification of microseismic records is urgently needed.

Throughout the years, many automatic classification methods have been proposed to address the abovementioned problems in seismic and microseismic fields. Scarpetta et al.^12^ established a specialized neural discrimination method for low magnitude seismic events, quarry blasts, underwater explosions, and thunder sources at Mt. Vesuvius Volcano, Italy. Langer^13^, Esposito^14^ and Curilem^15^ used the machine learning to classify seismic records at the Soufriere Hills volcano (Montserrat), Stromboli island (southern Italy) and the Villarrica volcano (Chile), respectively. Malovichko^16^ utilized a set of seismic characteristics and the multivariate maximum-likelihood Gaussian classifier, to quantify a probability that a particular event belongs to a population of blasts. Vallejos and McKinnon^17^ presented an approach to the classification of seismic records from two mines in Ontario, Canada, by using logistic regression approach and neural network classification techniques. Hammer et al.^18^ attempted to automatically classify seismic signals from scratch by utilizing a hidden Markov model and 30 features extracted from waveforms. Ma et al.^4^ realized the discrimination of mine microseismic events by Bayes discriminant analysis. Dong et al.^19,20^ proposed a discrimination method for seismic and blasting events based on a Fisher classifier, a naive Bayesian method and logistic regression; this method regards the logarithm of the seismic moment, the logarithm of the seismic energy, and the probability density function of the arrival time between adjacent sources as features.

Although these researches promote the research process in this field, it still cannot realize the automatic identification of complex microseismic records in the actual production process. In recent years, the deep learning approach has demonstrated superior performance in various research fields. Similarly, deep learning techniques are increasingly used in the field of seismology. Shang et al.^21^ established a classifier to distinguish microseismic records from quarry blasts by using Principal Component Analysis (PCA) and Artificial Neural Networks (ANN). ANN is considered the basis of the deep learning approach. Serdar Kuyuk and Ohno Susumu^22^ trained a deep learning Long Short-Term Memory (LSTM) network for the classification of near-source waveforms based on data from seismic events recorded by 305 three-component accelerometers recorded in Japan between 2000 and 2018. The LSTM network was tested with the earthquake in Northern Osaka (M 6.1) in 2018 as an example. Manuel Titos et al.^23^ proposed a novel approach in the field of volcano seismology to classify volcano-seismic events based on fully connected DNNs. The DNNs model was trained by 9,332 volcanic earthquake events to classify the seven types of events, and good experimental results were obtained. Bi Lin et al.^24^ proposes a method combining Convolutional Neural Networks (CNN) with Support Vector Machine (SVM) for identifying the multi-channel microseismic waveform automatically. They used 30,000 signal samples for CNN training, 3,960 event samples for SVM training, and finally achieved 98.18% classification accuracy. These new technologies and methods are encouraging because they effectively improve the accuracy and reliability of microseismic or seismic event classification.

However, the deep learning method requires a large amount of data to support the training process of its model. Hence, in actual applications, a large amount of manually labeled samples is required, which cannot be quickly applied in the newly built microseismic monitoring system of a mine, as the features of microseismic records in different mines vary greatly. Consequently, achieving a reliable real-time classification using limited samples is of great interest. Therefore, we concentrate on an approach with superior accuracy and stability to automatically classify multi-class microseismic records in underground mining using only limited samples. In this paper, we propose an approach to establish an automatic classifier for multi-class microseismic records with limited samples using the Capsule Network (CapsNet). This approach allows most of the current mines, both old and new, to use deep learning as early as possible to achieve the automatic classification of microseismic records and has a reliable result. The proposed method will be described in detail in the following sections. Subsequently, the proposed method will be applied to field datasets to demonstrate the efficiency and reliability of the classification of limited microseismic data.

## Results

**Figure 1:**
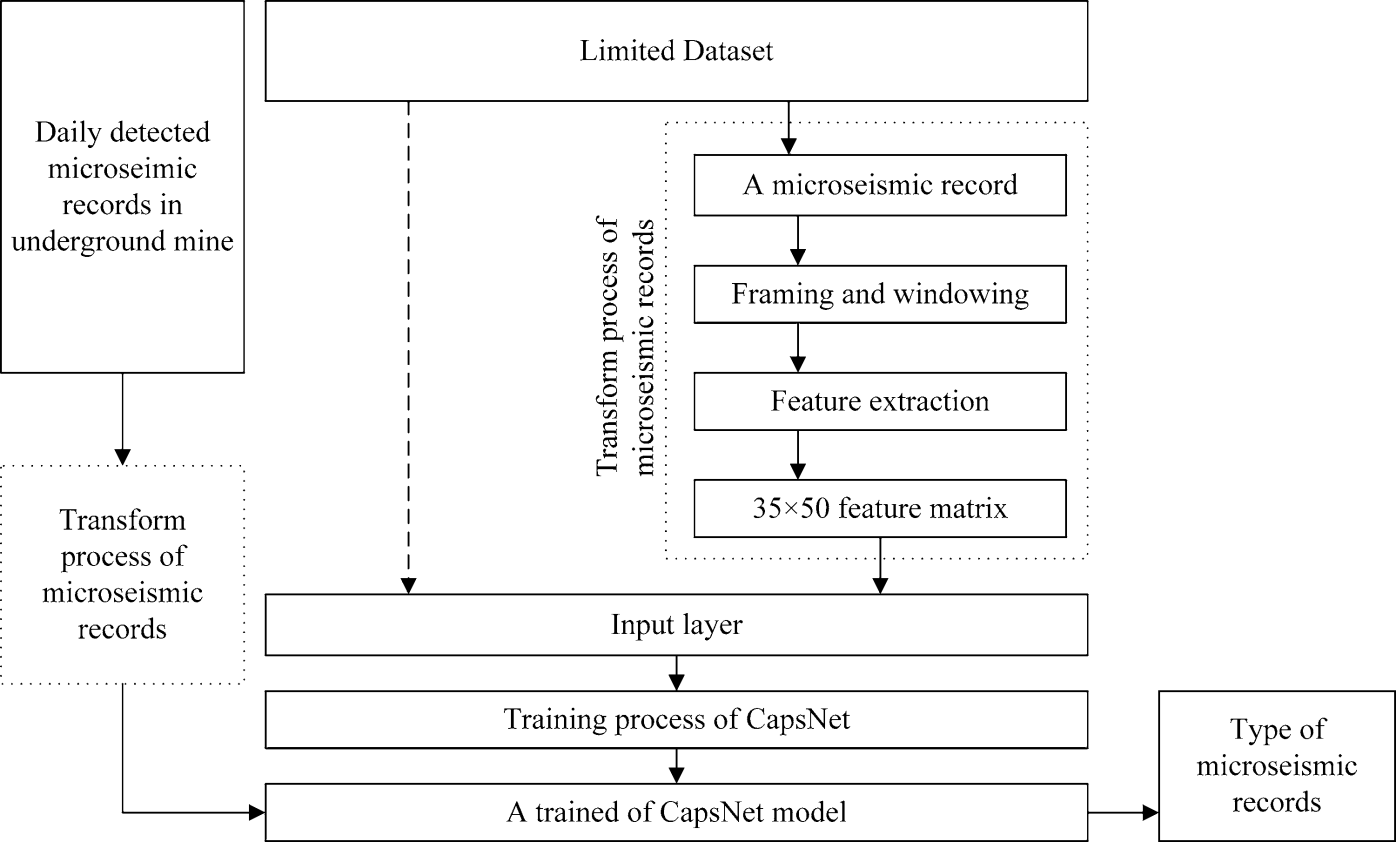
The actual application process of the automatic classification method in the mine.

we analyze and discuss the proposed method based on the actual application process of the automatic classification method. The accuracy and reliability of CapsNet, CNN and other methods are compared. Figure 1 shows the actual application process of the automatic classification method in the mine.

### Training process

Based on the microseismic records from the Huangtupo Copper and Zinc Mine, five training sets are divided according to different proportions, which contain 400, 800, 1,200, 1,600, and 2000 microseismic records. 20% of each training set will be used as the validation set. Moreover, one dataset had 3,200 microseismic records, 800 of each type, and no duplicate elements from the training were set as a universal test set. Training sets of different sizes constitute different training processes, and the situations of different training processes are shown in Table 1. The purpose of different training processes is to test the performance and reliability of CapsNet and CNN under limited samples.

**Table 1:** The situations of different training processes.

Process	Amount of data used for training	Amount of data used for validation	Amount of data used for the test	Distribution of different types of samples
Training process 1	320	80	3,200	Even
Training process 2	640	160	3,200	Even
Training process 3	960	240	3,200	Even
Training process 4	1,280	320	3,200	Even
Training process 5	1,600	400	3,200	Even

**Figure 2:**
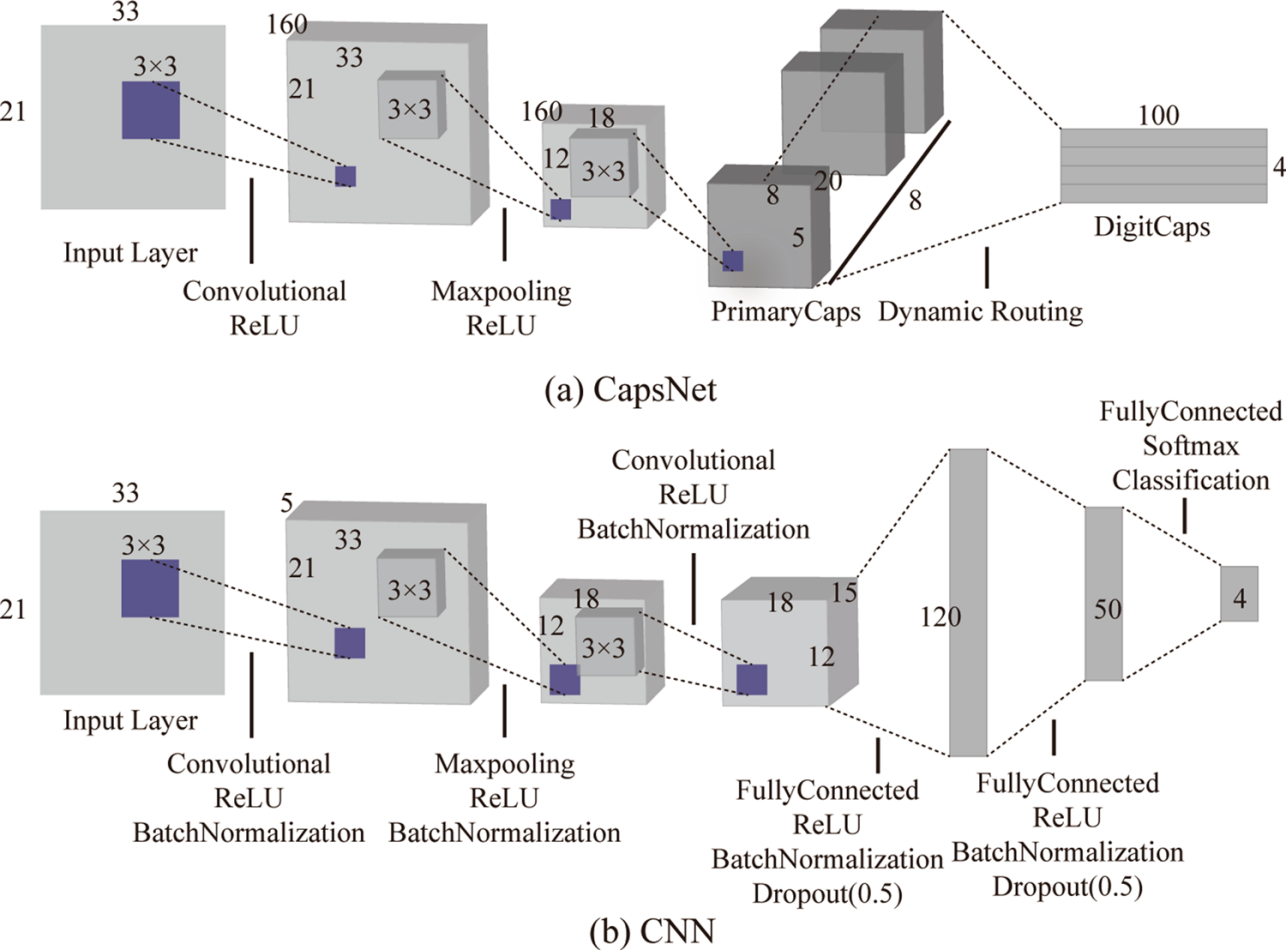
Detailed architecture and parameters of CapsNet and CNN.

**Figure 3:**
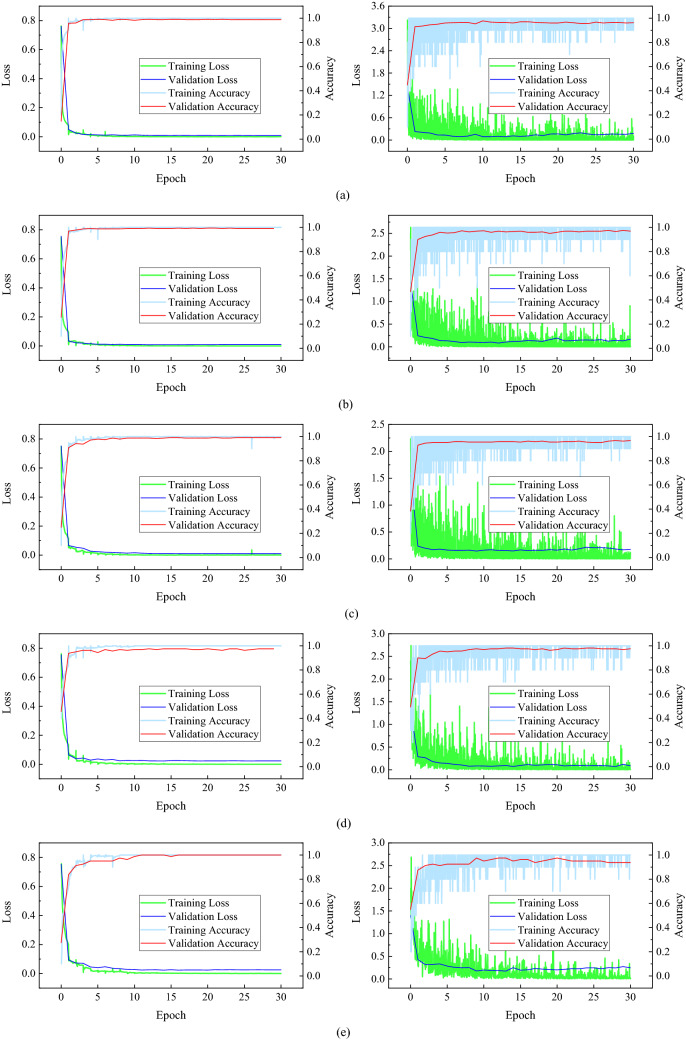
The training process of CapsNet and CNN. (**a**) is training process 5; (**b**) is training process 4; (**c**) is training process 3; (**d**) is training process 2; (**e**) is training process 1. the left column of the figure is CapsNet, and the right column is CNN.

With the parameters and architecture of the CapsNet and CNN showed in Fig. 2, we trained this two networks in different training processes (as shown in Table 1). The CapsNet consists of 2 convolution layers, a maxpooling layer, 2 ReLU layers, and a unique dynamic routing layer; the CNN consists of 2 convolution layers, a maxpooling layer, 5 ReLU layers, 5 batch normalization layers, 3 fully connected layers, 2 dropout layers, a softmax layer, and a classification layer. the minibatch size of all training process is 10 and ended in 30 epochs. The minibatch accuracy, validation accuracy, minibatch loss, and validation loss during the training process were recorded, and the training process were shown in Fig. 3.

From Fig. 3, the training process of CapsNet is stable and converges rapidly. Accuracy, loss, and validation curve closely match the training curve. However, for CNN, its training curve has been repeatedly beaten in 30 Epochs, eventually resulting in a low convergence state, even though it achieves higher accuracy. Through different training processes, we obtained five classification models of CapsNet and CNN, respectively.

### Accuracy and comparison

Based on the training process of the classification model, this section uses the test set to test the effect of these models. Moreover, the classification result of deep learning method is compared with the result of commonly used machine learning method. The test set consisted of 3,200 actual microseismic records of the Huangtupo Copper and Zinc Mine, with 800 records for each category, and none of these records appeared during the training and verification process. As an evaluation, Accuracy, Precision, Recall, and F1-Measure will be adopted^25^. Accuracy is the proportion of the microseismic record with the correct classification in test set:

1 $${\text{Accuracy}} = 1 - \frac{FP(tr) + FN(tr)}{{TP(tr) + TN(tr) + FP(tr) + FN(tr)}}$$

where *TP* denotes true positives (The records of the current type are correctly classified), *TN* denotes true negatives (The records of the other types are correctly classified), *FP* denotes false positives (The records of the other types are misclassified), and *FN* denotes false negatives (The records of the current type are misclassified). Precision is the proportion of predictions that are accurate, and Recall is the proportion of microseismic records that are correctly predicted:

2 $${\text{Precision } = \text{ }}\frac{TP(tr)}{{TP(tr) + FP(tr)}}$$

3 $${\text{Recall}} = \frac{TP(tr)}{{TP(tr) + FN(tr)}}$$

moreover, comprehensive considering Precision and Recall, the the weighted harmonic average evaluation index (F-Measure) has been proposed.

4 $${\text{F } - \text{ Measure}} = \frac{{(\alpha^{2} + 1) \times {\text{Precision}} \times {\text{Recall}}}}{{\alpha^{2} \times ({\text{Precision}} + {\text{Recall}})}}$$

when *α* = 1, is the most common F1-Measure:

5 $${\text{F1 } - \text{ measure}} = \frac{{2 \times {\text{Precision}} \times {\text{Recall}}}}{{{\text{Precision}} + {\text{Recall}}}}$$

**Figure 4:**
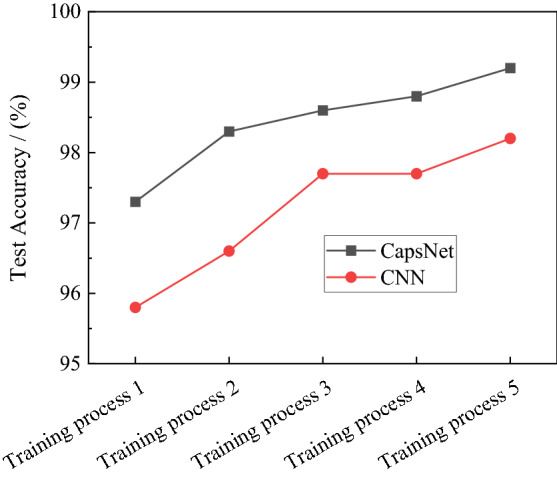
Accuracy of CapsNet and CNN in the different training process.

Figure 4 shows the test results of the trained CapsNet and the trained CNN, and these results demonstrate the accuracy of CapsNet is always higher than that of CNN. Taking into account more detailed comparisons, the abovementioned Precision, Recall, and F1-Measure are calculated for each type of microseismic records.

**Figure 5:**
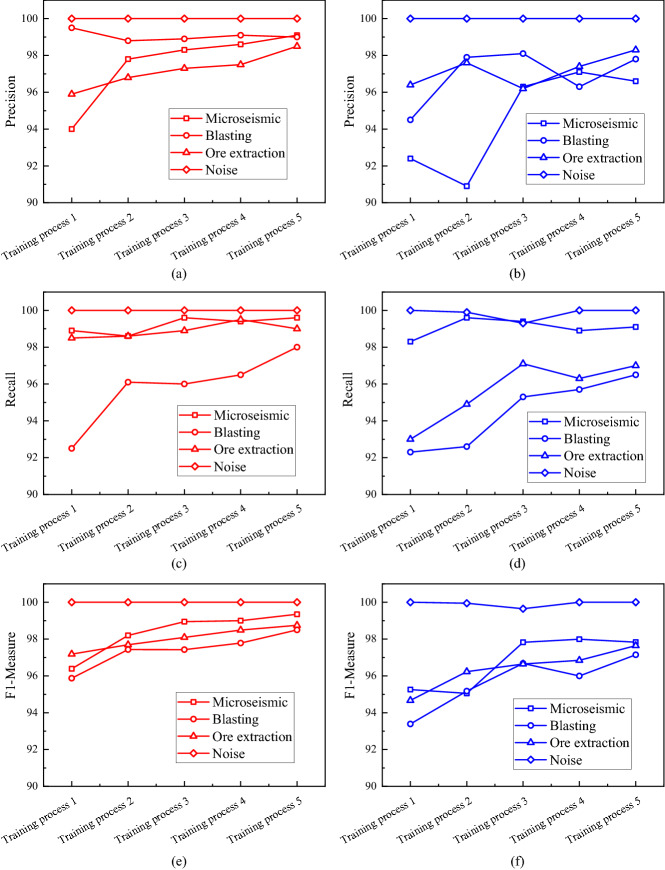
Comparison of Precision, Recall, and F1-Measure. (**a**) The precision of the CapsNet test results. (**b**) The precision of the CNN test results. (**c**) The Recall of the CapsNet test results. (**d**) The Recall of the CNN test results. (**e**) The F1-Measure of the CapsNet test results. (**f**) The F1-Measure of the CNN test results.

Figure 5a,b show the Precision of each type of microseismic records in the different training process. From Fig. 5a,b, the Precision of the CapsNet is much larger than the CNN’s in both types of microseismic and blasting records, and for both ore extraction and noise, the two are almost identical. It reveals that CapsNet's Precision is superior to CNN’s in different experiments. Also, Fig. 5c,d show the Recall of each type of microseismic records in the different training process. From Fig. 5c,d, the Recall of the CapsNet curve is much larger than the CNN in both types of blasting and ore extraction records, and for both microseismic and noise records, the gap still exists, but it is weak. It reveals that CapsNet's Recall is superior to CNN’s in different experiments. Through F1-Measure, we take a comprehensive consideration of the above two indicators. Figure 5e,f show the F1-Measure of each type of microseismic records in the different training process. It can be found that the value of CNN’s test results is always lower than the value of the CapsNet’s test results. Multiple indicators reveal that CapsNet has certain advantages over CNN in the classification of microseismic records.

Moreover, a comparison of the classification performance between the deep learning approach and traditional machine learning methods is presented. Decision tree and k-nearest neighbor (kNN) are often used to classify microseismic records. Therefore, we tested these models by utilizing the same dataset of training process 5 (details in Table 1) and compared their results with the findings from the deep learning approach proposed herein. Table 2 shows the classification results from different classification models, including the CapsNet and CNN presented in this paper, while utilizing the same dataset and features. For the testing accuracy, the CapNet performed the best. The testing accuracy of the CapsNet reached 99.2%, while the accuracies of the machine learning methods were below 90%. Each index of the CapsNet proposed in this paper outperformed those of the other methods. These findings demonstrate that the CapsNet has excellent efficiency and reliability for the classification of microseismic data.

**Table 2:** Comparison of different classification models.

Method	Accuracy /(%)	Microseismic			Blasting			Ore extraction			Noise		
		Precision/(%)	Recall/(%)	F1-Measure	Precision/(%)	Recall/(%)	F1-Measure	Precision/(%)	Recall/(%)	F1-Measure	Precision/(%)	Recall/(%)	F1-Measure
CapsNet	99.2	99.1	99.6	99.3	99.0	98.0	98.5	98.5	99.0	98.7	100.0	100.0	100.0
CNN	98.2	96.6	99.1	97.8	97.8	96.5	97.1	98.3	97	97.6	100.0	100.0	100.0
Decision tree	88.2	79.6	98.2	87.9	86.6	74.7	80.2	86.6	84.2	85.4	100.0	100.0	100.0
KNN	80.0	79.1	99.8	88.3	42.8	84.0	56.7	98.3	57.8	72.8	100.0	100.0	100.0

## Discussion and conclusion

Additionally, to show that CapsNet has clear advantages over CNN in microseismic records classification, we analyze the reliability of the two from the network classification probability. In deep learning, the final predicted output is composed of the decision probability of the corresponding labels, and the label corresponding to the maximum probability value is used as the predicted class of input. The probability used in this paper is the max probability of predicted output.

**Figure 6:**
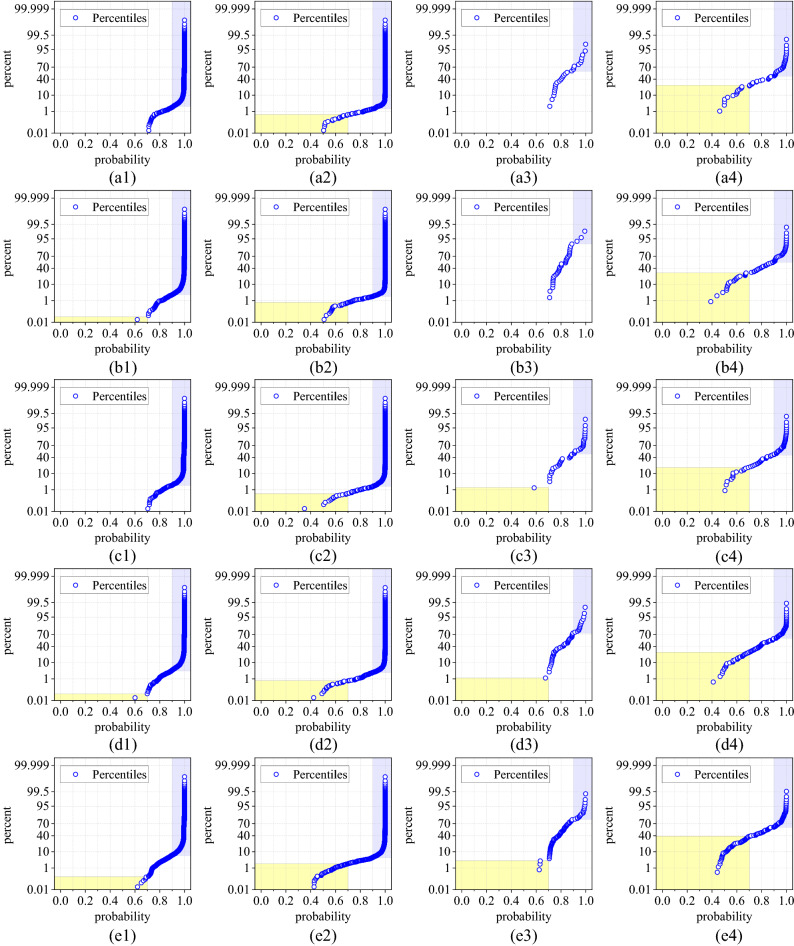
Distribution of classification probability in different situation. The labels (**a**)–(**e**) represent training process 5 to training process 1; the number 1 represent probability of each sample with correct classification results of CapsNet; the number 2 represent probability of each sample with correct classification results of CNN; the number 3 represent probability of each sample with incorrect classification results of CapsNet; the number 4 represent probability of each sample with incorrect classification results of CNN. For example, (**b1**) represent correct classification results of CapsNet in training process 2, but (**d4**) represent incorrect classification results of CapsNet in training process 4. Moreover, the light yellow blocks represent the probability value is below 0.70, but the light blue blocks represent the probability value is above 0.90.

Figure 6 shows the distribution of classification probability in different training process and classification results (correct and incorrect). For example, Fig. 6a1,a2 show the probability distribution of test samples that predicted class in keeping with the label after training process 5 by CapsNet and CNN. On the contrary, (a3) and (a4) show that of incorrect classification.

**Figure 7:**
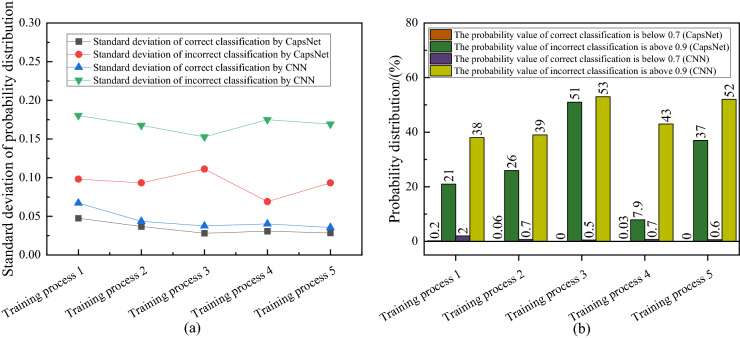
Detailed probability distribution comparisons. (**a**) The standard deviation of the probability distribution. (**b**) The proportion of probability value below 0.70 and above 0.90.

For the correct classification, the results of CapsNet is concentrated on higher probability value, which is almost always above 0.70, and there is a larger percentage of results below 0.70 for CNN. Moreover, for the incorrect classification, an excellent classifier should attribute the failure to the hesitant state, that is, the output probability of all types is similar and low. However, the results of CNN are concentrated on higher probability which is above 0.90, many samples are strongly misclassified. But CapNet's results are the opposite of CNN's. Detailed probability distribution comparisons are shown in Fig. 7. In summary, CNN's strong predictive characteristics for both correct and incorrect classifications result in lower reliability than CapsNet. CapsNet's strong prediction of correct classification and weak prediction of incorrect classification can effectively help inspectors to screen the results in specific situations.

**Figure 8:**
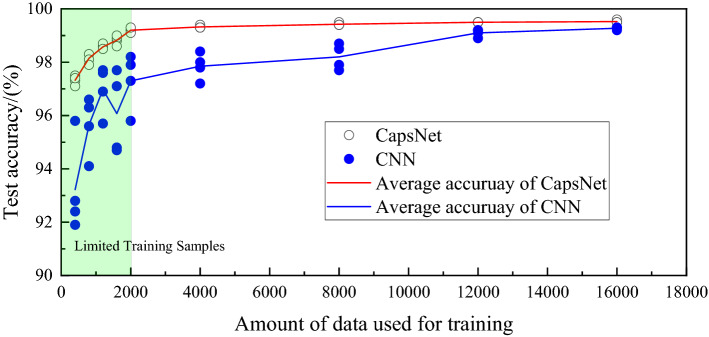
The results of repetitive experiments.

In order to more intuitively prove the advantages of CapsNet under limited data, we designed a set of repetitive experiments. We have prepared training sets with different amounts of data, which contain 400, 800, 1,200, 1,600, 2000, 4,000, 8,000, 12,000, and 16,000 microseismic records. We define the data volume less than 2000 as limited training samples. We perform four pieces of training and four tests on the model for each amount of data. As shown in Fig. 8, for each amount of data, we train four models for classification. From the experimental results, it can be seen that under limited training samples, CapsNet still has high accuracy and stability. However, for CNN, its accuracy is low, and the variation is large. As a consequence, CapsNet will outperform CNN in accuracy and stability for real applications with the everlasting scarcity issue of labeled seismic or microseismic data. Thus, CapsNet is a better option when we don’t have much labeled data at hand.

We propose a deep learning approach based on CapsNet to realize the automatic classification of microseismic records with limited samples in underground mining. CapsNet is a fully connected network of a series of interconnected capsules. In order to convert the microseismic record into the input for CapsNet, we extract the feature of the microseismic record by dividing a microseismic record waveform into 33 frames and extracting 21 feature parameters from each frame. Consequently, a 21 × 33 matrix is utilized to represent a microseismic record as the input of the CapsNet. On this basis, we use different sizes of training sets to train the classification models separately. The trained models are tested using the same test set containing 3,200 microseismic records and compared to CNN. Results show that CapsNet can achieve stable convergence faster than CNN with limited training samples. Then we use Accuracy, Precision, Recall, and F1-Measure as evaluation indexes. Results show that CapsNet is superior to CNN and traditional machine learning methods on various indicators. Finally, we analyze the reliability of the classification results of CapsNet and CNN. Results show that CapsNet performs better than CNN in terms of reliability. These results all indicate the reliability and practicability of CapsNet for automatic classification of microseismic records with limited samples in underground mining.

## Methods

### The principle of the CapsNet

At present, the deep learning architecture based on CNN architecture is widely used in various fields, such as image recognition, automatic driving, etc^26–28^. However, due to the convolution operation of CNN, only the existence information of the feature is retained in the recognition process, and the orientation of the feature and the spatial relationship are ignored. Moreover, the downsampling of the max-pooling layer discards much crucial information. Therefore, the conventional deep learning method represented by CNN requires much data for training^29^.

**Figure 9:**
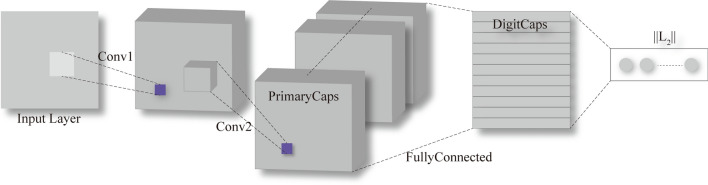
A network architecture for CapsNet, consists of three layers: two convolutional layers (Conv1 and Conv2) and one fully connected (FC)^30,31^.

The Capsule Network (CapsNet) represents an entirely novel type of deep learning architectures that attempt to overcome the abovementioned disadvantage of conventional deep learning. Figure 9 shows a typical architecture of CapsNet. The architecture is shallow with only two convolutional layers (Conv1 and Conv2 in Fig. 1) and one fully connected (FC) layer^30^. The outputs of each layer are Conv1d, Primary Capsule (PrimaryCaps), and Digit Capsule (DigitCaps). CapsNet was robust to the complex combination of features and required fewer training data. Also, CapsNet has resulted in some unique breakthroughs related to spatial hierarchies between features^32^. A capsule is a vector that can contain any number of values, each of which represents a feature of the object (such as a picture) that needs to be identified^33^. In CNN, each value of the convolutional layer is the result of a convolution operation. The convolution operation is a linear weighted summation, so the value of each convolutional layer is a scalar. However, in CapsNet, each value of the capsule is a vector; that is, this vector can represent not only the characteristics but also the direction and the state of the input.

**Figure 10:**
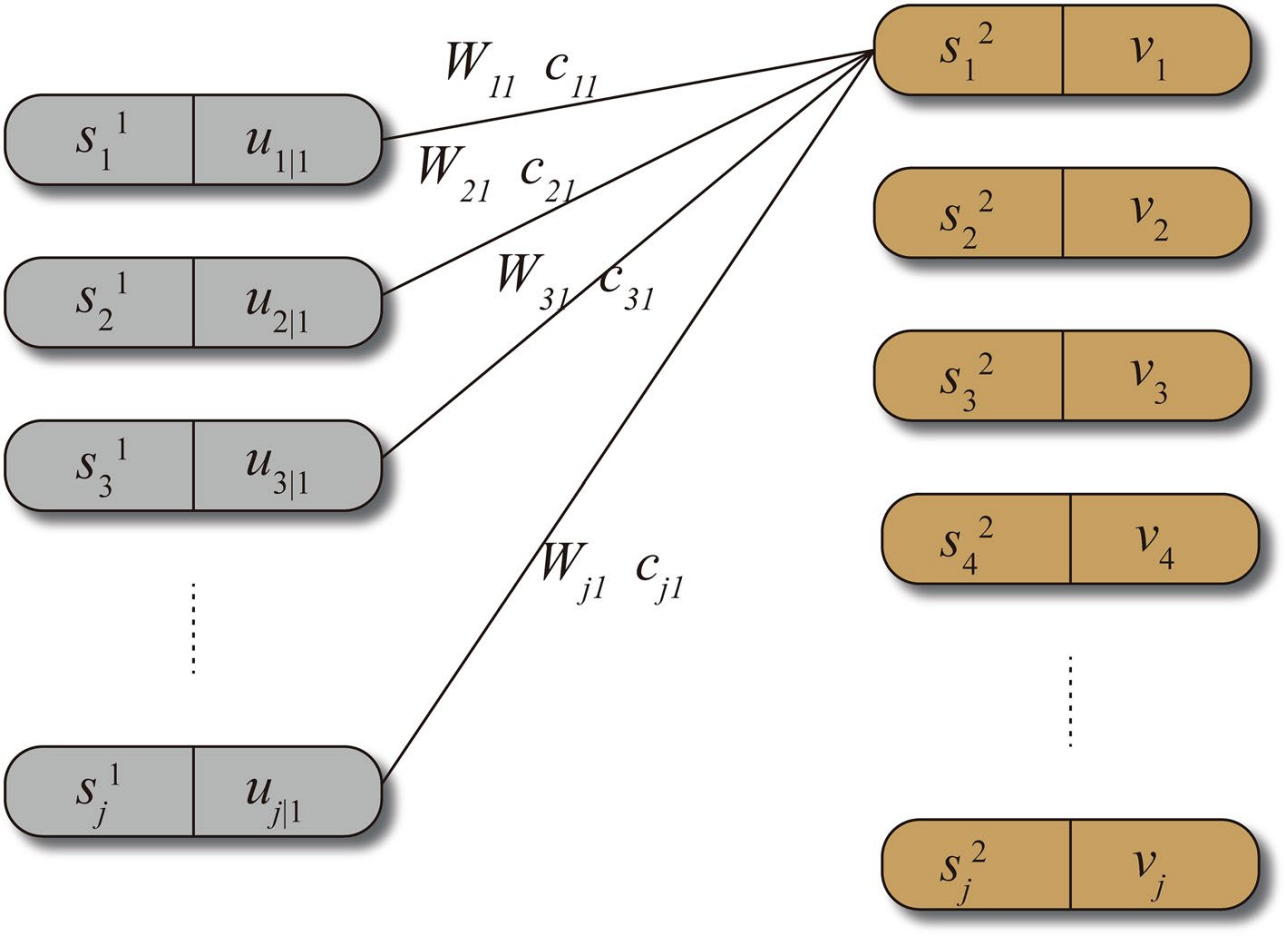
Dynamic routing algorithm that completes the transition from the PrimaryCaps layer to the DigitCaps layer.

Moreover, the CapsNet uses the dynamic routing algorithm to achieve data transmission between the capsule layers (as shown in Fig. 10), which overcomes the shortcomings of the traditional pooling layer^34^. In the dynamic routing algorithm, a non-linear "squashing" function (Eq. 6) is used to ensure that short vectors get shrunk to almost zero length and long vectors get shrunk to a length slightly below 1.

6 $${\text{v}}_{j} = \frac{{\left\| {s_{j} } \right\|^{2} }}{{1 + \left\| {s_{j} } \right\|^{2} }}\frac{{s_{j} }}{{\left\| {s_{j} } \right\|}}$$

where v_*j*_ is the vector output of the capsule *j* and *s*_*j*_ is its total input. And *s*_*j*_ is a weighted sum of all output ${\hat{\mathbf{u}}}_{j|i}$ of the previous layer. ${\hat{\mathbf{u}}}_{j|i}$ is produced by multiplying the output **u**_*i*_ with a weight matrix **W**_*ij*_.

7 $$s_{j} = \sum\limits_{i} {c_{ij} {\hat{\mathbf{u}}}_{j|i} }$$

8 $${\hat{\mathbf{u}}}_{j|i} = {\mathbf{W}}_{ij} {\mathbf{u}}_{i}$$

The *c*_*ij*_ in Eq. 7 denotes a coupling coefficient that is determined by the iterative dynamic routing process:

9 $$c_{ij} = \frac{{\exp (b_{ij} )}}{{\sum\nolimits_{k} {\exp (b_{ik} )} }}$$

where *b*_*ij*_ and *b*_*ik*_ are the log prior probabilities between two coupled capsules. Also, *b*_*ij*_ is in an ongoing process of updating:

10 $$b_{ij} \leftarrow b_{ij} + {\hat{\mathbf{u}}}_{j|i} {\text{v}}_{j}$$

The initial value of *b*_*ij*_ is 0. Therefore, in the forward propagation process of solving *s*_*j*_, we design weight matrix **W**_*ij*_ as random values, *b*_*ij*_ is initialized to 0 to get *c*_*ij*_, and dynamic update of *b*_*ij*_ continuously optimizes the coupling coefficient *c*_*ij*_. This series of calculations finally realized the dynamic routing propagation between the two layers of capsules^35^.

Except that the coupling coefficient *c*_*ij*_ is updated by dynamic routing, other convolution parameters of the entire network and **W**_*ij*_ in the CapsNet need to be updated according to the loss function:

11 $$L_{k} = T_{k} \max (0,m^{ + } - \left\| {v_{k} } \right\|)^{2} + \lambda (1 - T_{k} )\max (0,\left\| {v_{k} } \right\| - m^{ - } )^{2}$$

where *T*_*k*_ = 1, *m*^+^  = 0.9, and *m*^−^  = 0.1 by default. *λ* enables down-weighting of the loss for absent digit classes stops the initial learning from shrinking the lengths of the activity vectors of all the digit capsules^30^.

### Dataset

**Figure 11:**
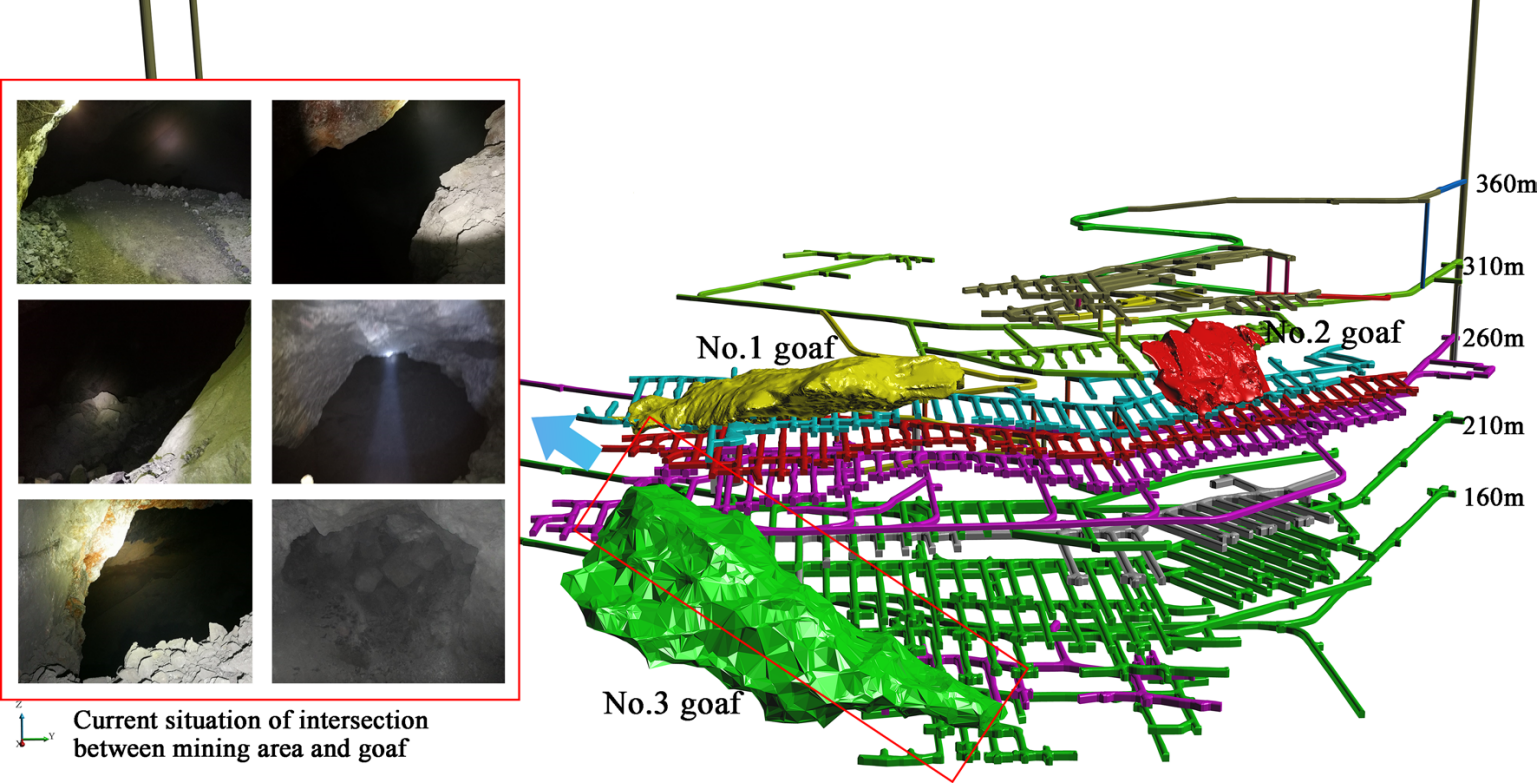
Distribution and influence of goaf in Huangtupo Copper and Zinc Mine.

The Huangtupo Copper and Zinc Mine is located in the southwest of Hami city, Xinjiang Uygur Autonomous Region, China. Two larger goaf areas (No.1 and No.2 goaf in Fig. 11) have been formed in this mine because of the use of non-pillar sublevel caving. Moreover, as the lower part and the upper part of the ore body are being mined at the same time, a larger and more unstable goaf area (No.3 goaf in Fig. 11) is formed at the mining junction. The volumes of these three goaves are 120,068.60 m^3^,42,633.25 m^3^, and 183,483.19 m^3^, respectively. Among them, the No.3 goaf area is much larger than the other two, and it is also the most dangerous. As shown in Fig. 11, No.3 goaf area has been interconnected with multiple mining routes, which is a severe crisis.

**Figure 12:**
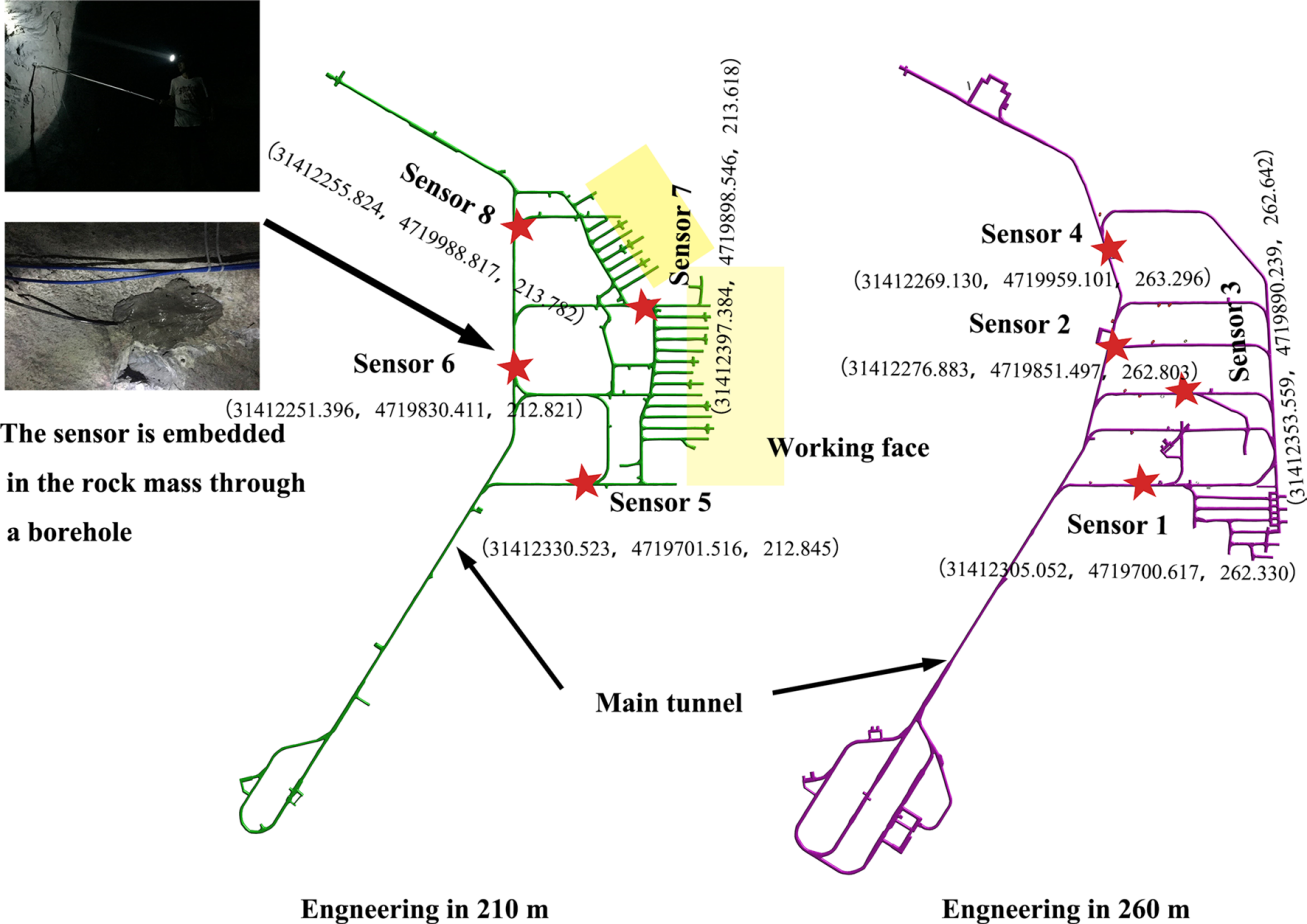
Coordinates of the accelerometers installed in the Huangtupo Copper and Zinc Mine.

To understand the stability of the rock mass, a microseismic system was used to perform continuous monitoring of around goaves and stopes. Eight single-component accelerometers with a sensitivity of 10 V/g and a sampling frequency of 10 kHz were embedded in the Huangtupo Copper and Zinc Mine. Their coordinates are shown in Fig. 12.

**Figure 13:**
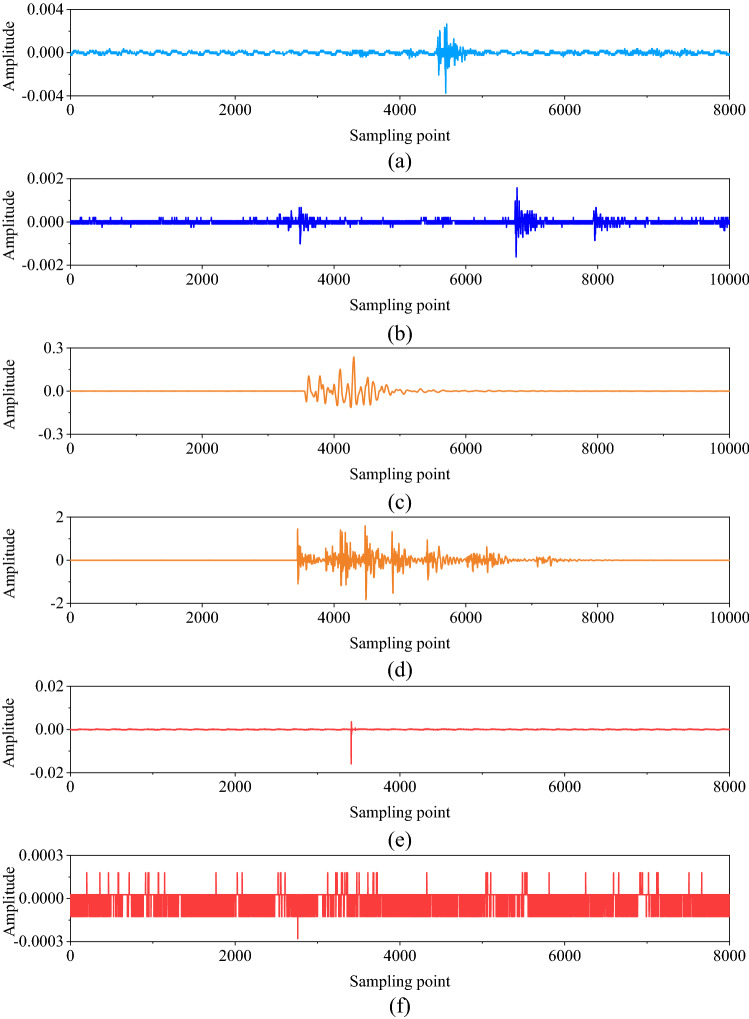
Example of microseismic records. (**a**) is a microseismic waveform, (**b**) is a waveform of ore-extraction event, (**c**) and (**d**) are the waveform of blasts, (**e**) and (**f**) are the waveform of instances of noise.

Hundreds of events are triggered in the Huangtupo Copper and Zinc Mine every day. Considering our processing goal to monitor rock activity and to provide early-warning systems, these events are categorized into four types: microseismic events, blasts, ore extraction, and noise. All events triggered between September 2017 and January 2019 were manually labeled and were selected as our dataset. The example of each type of event is shown in Fig. 13.

### Pretreatment

The original waveform is segmented every 380 sampling points to form a frame. A total of 80 points repeatedly appear between adjacent frames to avoid a large difference between adjacent frames. As a consequence, we can obtain 33 frames for each microseismic record under the condition that each record includes 10,000 sampling points. The purpose of waveform framing is to preserve the characteristics of the time sequence while transforming the waveform. Moreover, to maintain continuity between adjacent frames and attenuate the frequency leakage caused by signal truncation, each frame is multiplied by the Hamming window after the microseismic records are framed^10^. Assuming that the microseismic record is *S*(*n*), *n* = 1, 2, …, *N *− 1, multiplying the record by the Hamming window *w*(*n*) gives

12 $$S^{\prime}(n) = S(n) \times w(n)$$

where *w*(*n*) gives

13 $$w(n) = 0.54 - 0.46 \times \cos \left( {\frac{2\pi n}{{N - 1}}} \right), \, 0 \le n \le N - 1$$

where *N* is the number of frames within the framed microseismic record.

Then, we extract features of the time and frequency domains from each frame. Table 3 gives an overview of the 21 features employed most frequently in the literature for each frame used in this study. It is worth mentioning that these features are selected by the genetic algorithm (GA)-optimized correlation-based feature selection (CFS) method, for more detail implementation of feature selection, please see the reference^35, 36^. Zero-crossing rates are used to determine whether the microseismic record is present in a frame^37^. Energy and energy entropy can be used to indicate signal strength, and the strengths of different types of microseismic records show distinct differences^38^. The spectral centroid, spectral spread, spectral entropy, spectral flux, and spectral rolloff form the low-level spectral features, which aim to describe the structure of the frame spectra using a single quantity^39,40^; these features can be extracted within either linear or logarithmic frequency domains using spectral amplitudes, power values, logarithmic values, etc. Mel frequency cepstral coefficients (MFCCs) are an interesting variation on the linear cepstrum, which is widely used in signal analysis. MFCCs are the most widely used features in signal recognition, mainly due to their ability to concisely represent the signal spectrum^41,42^. Additionally, the harmonic ratio can be used to indicate the proportion of the signal composed of the non-microseismic record part^43^.

**Table 3:** Definitions and descriptions of features.

Num	Feature	Description	Definition	Parameters
1	Zero-Crossing Rate^37^	The rate of sign changes within a signal	$zcr = \frac{1}{T - 1}\sum\limits_{t = 1}^{T - 1} {II\{ s_{t} s_{t - 1} < 0\} }$	***s*** is a signal of length *T;* the indicator function $II\{ A\}$ is 1 if its argument *A* is true and 0 otherwise
2	Energy^38^	The energy of the waveform	$E = \sum\limits_{t = 1}^{T} {\left| {s_{t} } \right|^{2} }$	***s*** is a signal of length *T*
3	Energy Entropy^38^	The entropy of the energy of each frame	$EE = - \sum\limits_{n} {\frac{{E_{n} }}{E}} \ln \frac{{E_{n} }}{E}$	*E* is the total energy of a signal; *E*_*n*_ is the energy of the frame
4	Spectral Centroid^39^	The centre of the spectral density function	$SC_{i} = \frac{{\sum\nolimits_{j = 0}^{N - 1} {f_{i} (j)E_{i} (j)} }}{{\sum\nolimits_{j = 1}^{N - 1} {E_{i} (j)} }}$	*N* is the length of the signal; *f*_*i*_(*j*) is the frequency of the *j-*th point of the *i-*th frame; *E*_*i*_(*j*) is the spectral energy of the corresponding frequency of the *i-*th frame
5	Spectral Spread^39^	A measure of the average spread of the spectrum in relation to its centroid	$SS = \sqrt {\frac{{\sum\nolimits_{i = 0}^{N/2} {(f_{k} - SC)^{2} \left| {X(w_{i} )} \right|^{2} } }}{{\sum\nolimits_{i = 0}^{N/2} {\left| {X(w_{i} )} \right|^{2} } }}}$	*X*(*w*_*i*_) is the spectrum of the signal
6	Spectral Entropy^39^	The complexity of a signal	$PSE = - \sum\limits_{i = 1}^{n} {\frac{{\frac{1}{N}\left| {X(w_{i} )} \right|^{2} }}{{\sum\nolimits_{i} {\frac{1}{N}\left| {X(w_{i} )} \right|^{2} } }}} \ln \left( {\frac{{\frac{1}{N}\left| {X(w_{i} )} \right|^{2} }}{{\sum\nolimits_{i} {\frac{1}{N}\left| {X(w_{i} )} \right|^{2} } }}} \right)$	*X*(*w*_*i*_) is the spectrum of the signal
7	Spectral Flux^39^	A measure of how quickly the power spectrum of a signal is changing	$SF = \sum\limits_{i = 1}^{N} {[\left| {\hat{X}(w_{i} )} \right| - \left| {\hat{X}_{preframe} (w_{i} )} \right|]^{2} }$	$\hat{X}(w_{i} )$ is the normalized spectrum of the signal
8	Spectral Rolloff^39^	The frequency below which 90% of the magnitude distribution of the spectrum is concentrated	$\mathop {\arg \min }\limits_{{f_{c} \in \{ 1,...,N\} }} \sum\limits_{i = 1}^{{f_{c} }} {m_{i} \ge 0.90 \cdot \sum\limits_{i = 1}^{N} {m_{i} } }$	*f*_*c*_ is the rolloff frequency, and *m*_*i*_ is the magnitude of the *i*-th frequency component of the spectrum
9–20	Mel frequency cepstral coefficient^41,42^	A representation of the short-term power spectrum of a signal based on a linear cosine transform of a log power spectrum on a nonlinear Mel scale of frequency	$MFCC(n) = \sum\limits_{m = 0}^{M - 1} {\left[ {\ln (\sum\limits_{i = 1}^{N - 1} {\left| {X(i)} \right|^{2} H_{m} (i)} ) \cdot \cos (\frac{\pi n(m - 0.5)}{M})} \right]}$	*MFCC*(*n*) is the *n*-th Mel frequency cepstral coefficient, *n* = 2, …, 13; *X*(*i*) is the spectrum of the signal; *H*_*m*_(*k*) denotes the *M* filter banks, 0 ≤ *m* < *M*; *N* is the number of frame points
21	Harmonic Ratio^43^	A feature reflecting the ratio of energy in the harmonic portion of the signal to the total energy of the signal	$HR = \mathop {\max }\limits_{{M_{0} \le m \le M}} \{ \frac{{\sum\limits_{n = 1}^{N} {s(n)s(n - m)} }}{{\sqrt {\sum\limits_{n = 1}^{N} {s(n)^{2} \sum\limits_{n = 0}^{N} {s(n - m)^{2} } } } }}\} ,1 \le m \le M$	*s* is a single frame of a signal with *N* points; *M* is the maximum lag in the calculation; *M*_0_ denotes the first zero crossings of the normalized autocorrelation

**Figure 14:**
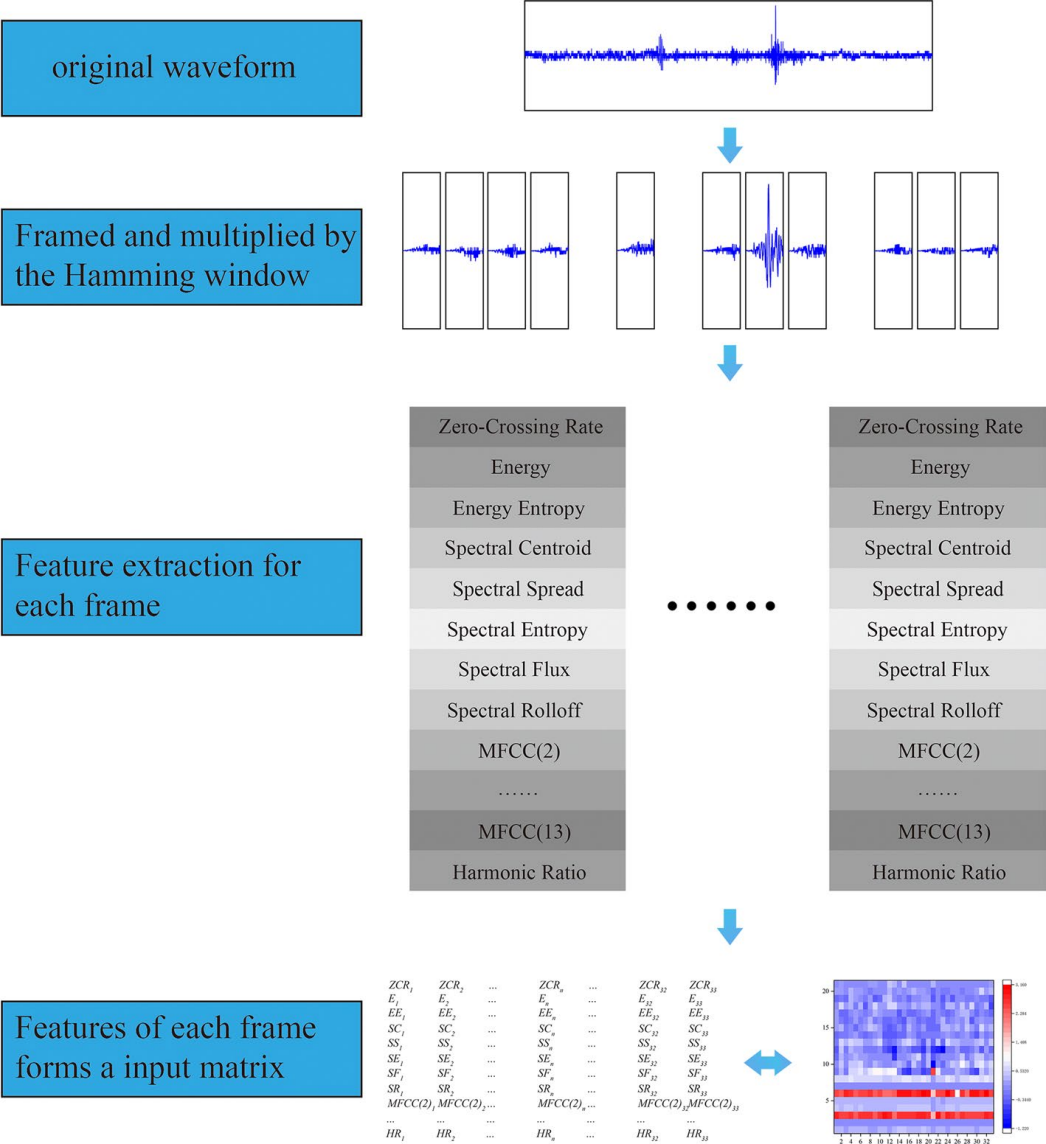
The process of converting the microseismic waveform into the available input to the capsule network.

As a consequence, a microseismic record is transformed into a 21 × 33 feature matrix by framing and feature extraction. Figure 14 shows the process and result of the transform. This 21 × 33 feature matrix is the initial input of the CapsNet.
